# Effect of pemafibrate in reducing intestinal long-chain fatty acid absorption and hepatic fibrosis in metabolic dysfunction-associated steatohepatitis rats

**DOI:** 10.1186/s12876-025-03967-z

**Published:** 2025-05-19

**Authors:** Masaya Okada, Masakazu Hanayama, Yasunori Yamamoto, Teruki Miyake, Osamu Yoshida, Eiji Takeshita, Yoshio Ikeda, Yoichi Hiasa

**Affiliations:** 1 Department of Gastroenterology and Metabology, Ehime University Graduate School of Medicine, Toon, Ehime Japan; 2 Department of Gastroenterology, Matsuyama Shimin Hospital, Matsuyama, Ehime Japan; 3 Endoscopy Center, Ehime University Hospital, 454 Shitsukawa, Toon, Ehime 791-0295 Japan; 4 Department of Inflammatory Bowel Diseases and Therapeutics, Ehime University Graduate School of Medicine, Toon, Ehime Japan

**Keywords:** CD36, Microsomal triglyceride transfer protein, Triglycerides, Lipotoxicity, Peroxisomes, Pemafibrate

## Abstract

**Background:**

Pemafibrate helps regulate fatty acid dynamics in the liver, potentially preventing metabolic dysfunction-associated steatohepatitis (MASH). However, its effect on intestinal long-chain fatty acid (LCFA) metabolism in MASH remains unclear. Thus, we aimed to examine the influence of pemafibrate on intestinal LCFA metabolism and hepatic fibrosis in a MASH rat model.

**Methods:**

Sprague–Dawley rats were fed a high-fat and high-cholesterol diet to induce MASH and then divided into pemafibrate-treated (pemafibrate (+)) and untreated (pemafibrate (-)) groups. Triglyceride deposition in the small intestine and fibrosis, along with α-smooth muscle actin level in the liver, were evaluated. Furthermore, the mRNA expression levels of genes associated with lipid metabolism in the small intestine and markers of fibrosis and hepatic stellate cells activation in the liver were measured.

**Results:**

The pemafibrate-treated group had markedly lower triglyceride deposition and lipid absorption in the intestine, and significantly lower levels of molecules involved in intestinal lipid regulation than the pemafibrate-untreated group. Moreover, hepatic fibrosis significantly improved, and the mRNA levels of fibrosis-related molecules and hepatic stellate cell activation factors significantly decreased in the pemafibrate-treated compared with those in the pemafibrate-untreated group.

**Conclusions:**

Pemafibrate reduced lipid droplet formation and LCFA absorption in the intestinal tract and alleviated hepatic fibrosis in MASH model rats.

## Background

Metabolic dysfunction-associated steatotic liver disease (MASLD), previously known as non-alcoholic fatty liver disease (NAFLD), is a chronic condition with a poor prognosis, often progressing to fibrosis, cirrhosis, and hepatocellular carcinoma [1–7]. In recent years, the incidence of cirrhosis and hepatocellular carcinoma has rapidly increased in the context of metabolic dysfunction-associated steatohepatitis (MASH), previously known as non-alcoholic steatohepatitis (NASH) [7]. Saturated fatty acids (SFAs) play a central role in the pathogenesis and progression of MASH, contributing to hepatic steatosis, inflammatory cytokine induction, and hepatocarcinogenesis [8, 9]. Among these, palmitic acid, a representative of long-chain fatty acids (LCFAs), exerts lipotoxicity and exacerbates MASH development by interacting with enteric endotoxin [10, 11].

The primary sources of SFAs for hepatocytes include hepatic de novo synthesized FAs, visceral adipose tissue, and dietary FAs absorbed from the small intestine [12]. Patients with MASH have a higher small intestinal capacity to absorb palmitate, with absorption levels positively correlating with markers of hepatic fibrosis, than healthy individuals [13]. Furthermore, cluster of differentiation 36 (CD36) and microsomal triglyceride transfer protein (MTP), which are intestinal molecules critical for dietary FA absorption and chylomicron synthesis, are overexpressed in the small intestine of patients with MASH [13, 14]. These findings suggest that modulation of dietary LCFAs in the small intestine could serve as a potential therapeutic target for MASH.

Peroxisome proliferator-activated receptor α (PPARα) is a nuclear receptor that regulates hepatic lipid homeostasis and influences MASH progression [15]. It is expressed in various tissues, including the liver, small intestine, heart, kidney, muscle, and brown adipose tissue [16–18]. Notably, global *Pparα*-knockout mice exhibit exacerbated MASH but not insulin resistance, highlighting the complexity of the role of PPARα [19]. Moreover, PPARα activation has been associated with increased satiety and decreased intestinal cholesterol esterification [20, 21]. However, the specific effects of intestinal PPARα inhibition on obesity and MASH remain controversial. In clinical practice, PPARα agonists, such as fibrates, are commonly used to treat dyslipidemia [22]. However, their use in the treatment of human MASH has not been established.

Pemafibrate is a selective PPARα modulator that reduces fibrate-related adverse events, such as rhabdomyolysis, while enhancing therapeutic effects [23]. It also reportedly improved hepatic steatosis and fibrosis in a rodent model of MASH [24]. However, the effect of pemafibrate on intestinal LCFA absorption in MASH models remains unclear. Therefore, in the present study, we aimed to investigate the effect of pemafibrate on intestinal LCFA absorption in a diet-induced MASH rat model and evaluate its therapeutic potential against hepatic fibrosis. This study could provide novel insights into the effects of pemafibrate on intestinal LCFA absorption and hepatic fibrosis in MASH model rats.

## Methods

### Animals

This study was carried out in strict accordance with the recommendations in the guidelines of Ehime University (Ehime, Japan). The protocol was approved by the Ehime University Animal Care Committee (Protocol Number: 05-TI-86-2). Eight-week-old male Sprague–Dawley (SD) rats (CLEA Japan, Tokyo, Japan) were housed individually and maintained under a 12:12-h light/dark cycle. All rats were acclimated to a normal MF diet (MF; Oriental Yeast Industry, Tokyo, Japan) (12.4% of kcal fat) and water for 7 days before the start of the experiment. They were then fed a high-fat and high-cholesterol diet (HFHCD; 68% MF, 27.5% palm oil, 2.5% cholesterol, and 2% cholic acid) (52.9% of kcal fat) for 9 weeks as previously described [25]. The rats were randomly assigned to two groups; pemafibrate (+) and pemafibrate (-), that is pemafibrate-treated and pemafibrate-untreated, and administered the treatment by oral gavage. The rats in the pemafibrate-treated group were administered pemafibrate (0.1 mg/kg/day) and olive oil (1 g/kg/day) into the stomach using a sonde once a day at a fixed time. The rats in the pemafibrate-untreated group (control) received vehicle instead of pemafibrate while maintaining the same conditions mentioned above. Oral gavage was performed with the rats under isoflurane anesthesia and euthanasia was performed using carbon dioxide. Pemafibrate was obtained from Kowa Co., Ltd. (Tokyo, Japan). All rats were provided water *ad libitum* during the experiment. All rats were randomly assigned to each group. Histological and gene expression analyses were performed in a blinded manner to minimize bias. For all histological assessments, tissue samples were coded and independently evaluated by an investigator who was blinded to the groups. Experiments were initially conducted using *N* = 5 rats per group. However, samples were excluded from the gene expression analysis of intestinal tissue due to a technical issue encountered during RNA extraction (poor quality yield). To ensure data accuracy, the samples was removed from this specific analysis, resulting in *N* = 4 for the intestinal gene expression data. All other analyses presented were performed using *N* = 5 per group. This sample exclusion was due to technical reasons and was not biased towards any specific treatment group.

### Tissue sample Preparation

All rats were fasted overnight and then euthanized under deep anesthesia. The liver and jejunal tissues were collected and preserved as follows. One section was fixed in 10% formalin for 24 h and embedded in paraffin, whereas the other was soaked overnight in RNA-later (Life Technologies, Carlsbad, CA, USA) and stored at − 20 °C until use.

### Measurement of intestinal lipid absorption coefficient

For each rat, 24-h food intake was recorded and fecal samples were analyzed. All rats in both groups were fed the same MF normal diet during the stool collection period. Lipids in the stool and diet were extracted using the Bligh–Dyer method and quantified. Briefly, chloroform (Wako Pure Chemicals, Tokyo, Japan) and methanol (Fujifilm, Tokyo, Japan) were added to the samples at a 1:2 ratio, and the layers were separated via centrifugation. The lower layer was carefully collected, and the chloroform was dried to extract the lipids. The lipid absorption coefficient (LAC) was calculated as follows:

1 $$LAC = \frac{dietary\ lipid\ intake - lipid\ defecation}{dietary\ lipid\ intake} \times 100$$

### Identification of genes involved in lipid and FA absorption via real-time polymerase chain reaction

The expression of genes involved in lipid and FA absorption in the jejunal tissue was measured using real-time polymerase chain reaction (PCR). RNA was extracted from the jejunum and liver samples using an RNeasy Plus mini kit (Qiagen, Hilden, Germany). Reverse transcription was performed using the High Capacity cDNA reverse transcription kit (Thermo Fisher Scientific, Waltham, MA, USA). Real-time PCR was performed using LightCycler 480II (Roche Diagnostics, Basel, Switzerland). The sequence of primers is presented in Table 1. Gene expression was normalized to that of hypoxanthine phosphoribosyltransferase 1 (*Hprt1*) in the liver and β-actin (*Actb*) in the jejunum. They are expressed as a ratio of the values obtained in the pemafibrate-untreated (control) group.

**Table 1:** Oligonucleotide sequences and annealing temperature for quantitative real-time PCR

Gene name		Sequence (5ʹ–3ʹ)	Annealing Temperature (°C)
*Ppara*	forward	CCTCGAACTGGATGACAGTG	59
	reverse	CCCTCCTGCAACTTCTCAAT	
*Cd36*	forward	GCGACATGATTAATGGCACA	60
	reverse	TGGACCTGCAAATGTCAGAG	
*Mtp*	forward	GCGAGTCTAAAACCCGAGTG	59
	reverse	CACTGTGATGTCGCTGGTTATT	
*Fabp1*	forward	ACTGGGGAAAAGGTCAAGGC	59
	reverse	CCCAGTGTCATGGTATTGGTGAT	
*Fapb2*	forward	CCGAGAGATTTCTGGTAACGA	59
	reverse	CAAGCTAGCCCTTCTGCATT	
*Fatp4*	forward	ATGACTGCCTCCCCCTCTAC	59
	reverse	AGTCATGCCGTGGAGTACG	
*Apoa-IV*	forward	ACCCAGCTAAGCAACAATGC	59
	reverse	AAGTTTGTCCTGGAAGAGGGTA	
*Actab*	forward	CTGGCTCCTAGCACCATGA	61
	reverse	TAGAGCCACCAATCCACACA	
*Tgfb*	forward	CCTGGAAAGGGCTCAACAC	59
	reverse	TGCCGTACACAGCAGTTCTT	
*Col1a1*	forward	GTGGACAGGCTGGTGTGAT	59
	reverse	GGGACACCTCGTTCTCCAG	
*Acta2*	forward	GCTCCGGGCTCTGTAAGG	59
	reverse	GCCCATTCCAACCATCACT	
*Timp1*	forward	TGCAACTCGGACCTGGTTAT	59
	reverse	AGCGTCGAATCCTTTGAGCA	
*Serpine1*	forward	AGAGCCAATCACAAGGCACT	59
	reverse	GAGGCAAGTGAGGGCTGA	
*Hprt1*	forward	TCCTCATGGACTGATTATGGACA	60
	reverse	TAATCCAGCAGGTCAGCAAAGA	

### Histological and morphometric analyses

The formalin-fixed and paraffin-embedded rat liver tissue and jejunum samples were used for the analyses. The liver tissues were stained with hematoxylin and eosin (H&E), and then with Sirius Red to assess liver fibrosis. Immunostaining was performed using polyclonal α-smooth muscle actin (α-SMA) (# RB-9010; 1:200; Thermo Fisher Scientific) as the primary antibody and MAX-POI (#414181; Nichirei Co., Tokyo, Japan) as the secondary antibody. After immunostaining, color was developed using 3,3ʹ-diaminobenzidine chromogen, and the tissues were evaluated. SR- or α-SMA-positive areas were measured using light microscopy. The jejunum samples were stained with Oil Red O to evaluate the degree of fat deposition.

### Statistical analysis

Results are presented as mean ± standard deviation. The results were analyzed using Mann–Whitney *U*-test. All statistical analyses were performed using JMP version 11.2.0 software (SAS Institute, Cary, NC, USA). Statistical significance was considered at *P* < 0.05.

## Results

### Changes of postprandial fatty acid absorption in the intestine of MASH model rats upon pemafibrate treatment

**Fig. 1:**
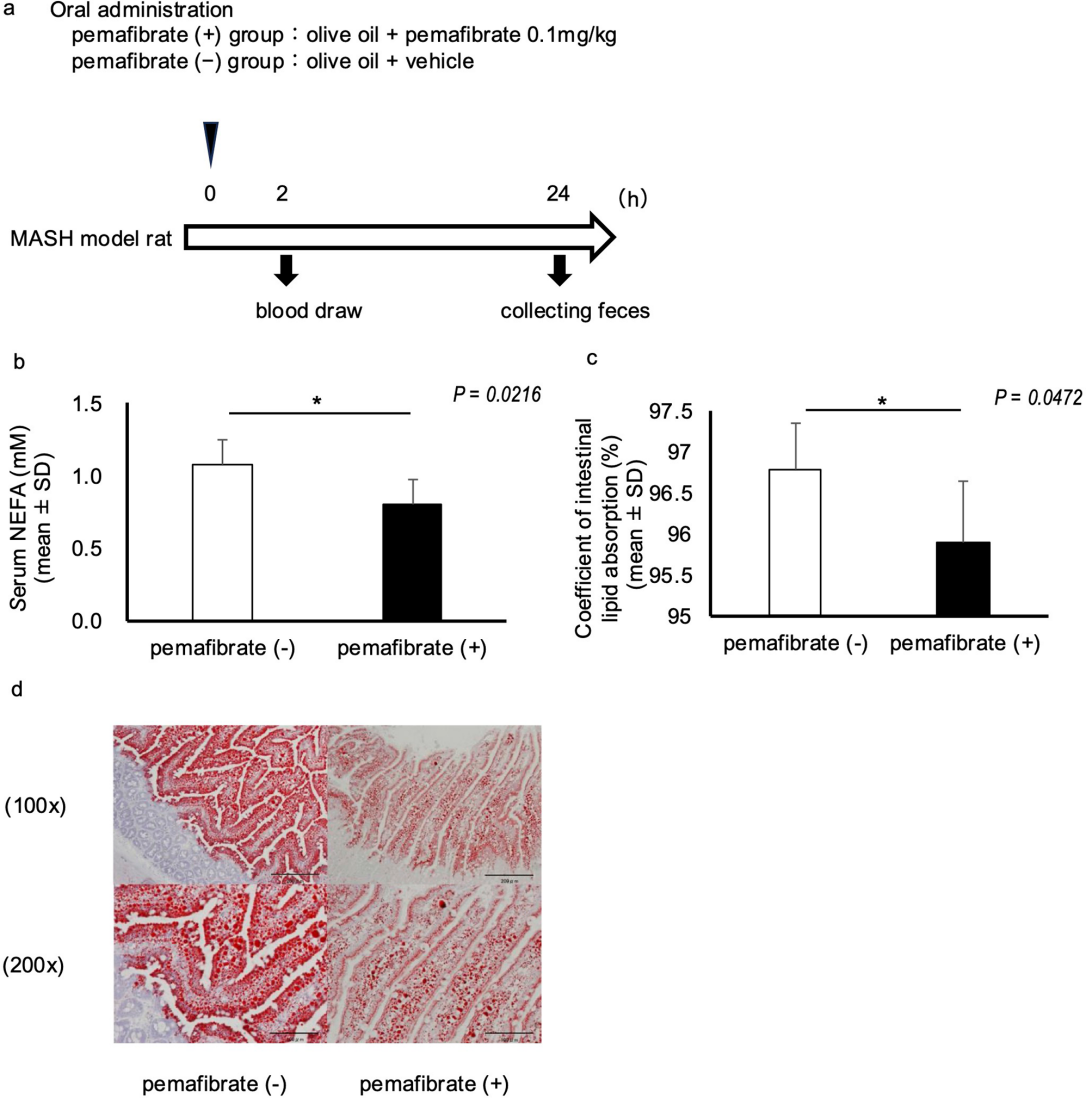
Pemafibrate inhibits postprandial fatty acid absorption in the small intestine of MASH model rats. (**a**) Experimental protocol. To evaluate postprandial intestinal fat absorption, a single dose of pemafibrate or vehicle was administered with olive oil, and fecal samples were collected 24 h later. (**b**) Serum NEFA levels in the pemafibrate (-) and pemafibrate (+) groups. (**c**) Coefficient of intestinal lipid absorption in the pemafibrate (-) and pemafibrate (+) groups. *N* = 5 per group; *P < 0.05. Data in the bar plots are expressed as mean ± standard deviation. Significant differences were determined using the Mann–Whitney U-test. (**d**) Representative images of the jejunum tissue stained with Oil Red O in the pemafibrate (-) and pemafibrate (+) groups. Top: magnification, 100×; scale bar, 200 μm. Bottom: magnification, 200×; scale bar: 100 μm (bottom)

We fed male Sprague–Dawley (SD) rats a HFHCD to generate an MASLD/MASH model. For the evaluation of postprandial intestinal fat absorption, a single dose of pemafibrate or vehicle was administered with olive oil, blood was drawn 2 h later, and fecal samples were collected 24 h later (Fig. 1a). The serum level of non-esterified fatty acid (NEFA) was significantly lower in the pemafibrate-treated group than in the pemafibrate-untreated group (*P* = 0.0216; Fig. 1b). Measurement of the amounts of fat in the diet over a 24-h period and fat excreted in the feces indicated that the fat-absorption capacity was significantly lower in the pemafibrate-treated group than in the pemafibrate-untreated group (*P* = 0.0472; Fig. 1c). Moreover, histological analysis of fat deposition in the jejunum with Oil Red O staining revealed that fat deposition in the small intestinal epithelium was considerably lower in the pemafibrate-treated group than in the pemafibrate-untreated group (Fig. 1d).

Pemafibrate treatment resulted in greater fat content remaining within the intestinal tract, consistent with reduced absorption, whereas intestinal tissue lipid accumulation was lower than in controls.

### Expression of PPARα and molecules involved in the absorption and transport of FAs in the intestine of MASH model rats

**Fig. 2:**
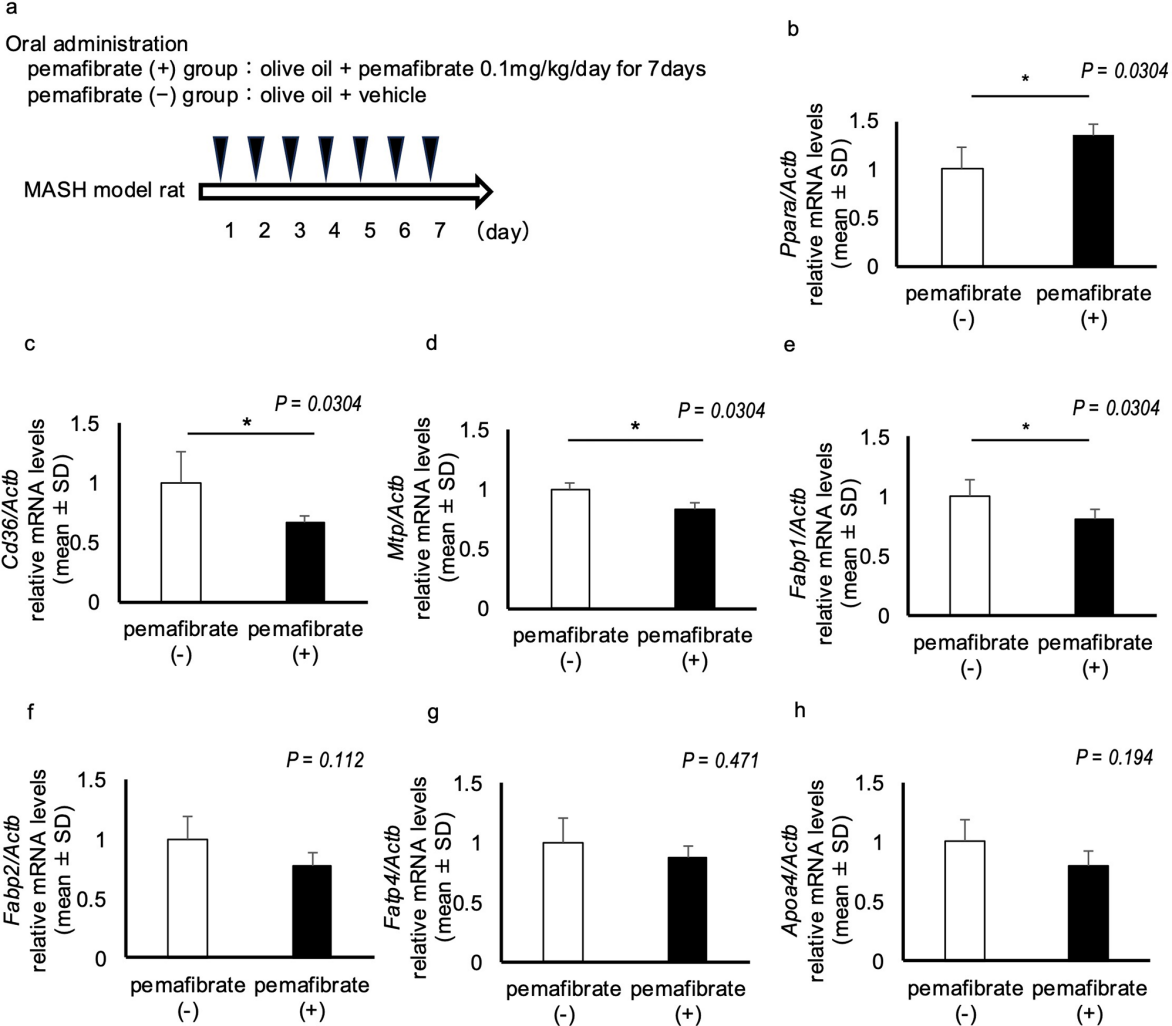
Expression of genes involved in fatty acid and lipid absorption in the intestine. (**a**) Experimental protocol. Pemafibrate or vehicle was administered with olive oil to rats for 7 days, and then the rats were dissected. (**b**) The mRNA level of *Ppara*. *N* = 4 per group; *P < 0.05. (**c-h**) The mRNA levels of genes related to the transport of long-chain fatty acids and lipids, c; cluster of differentiation (Cd36), d; microsomal triglyceride transfer protein (Mtp) and fatty acid-binding protein (Fabp), including e; liver FABP (Fabp1) and f; intestinal FABP (Fabp2), g; fatty acid transporter protein 4 (Fatp4), and h; apolipoprotein A-IV (Apoa-IV). *N* = 4 per group; *P < 0.05. Data in the bar plots are expressed as mean ± standard deviation. Significant differences were determined using the Mann–Whitney U-test

**Fig. 3:**
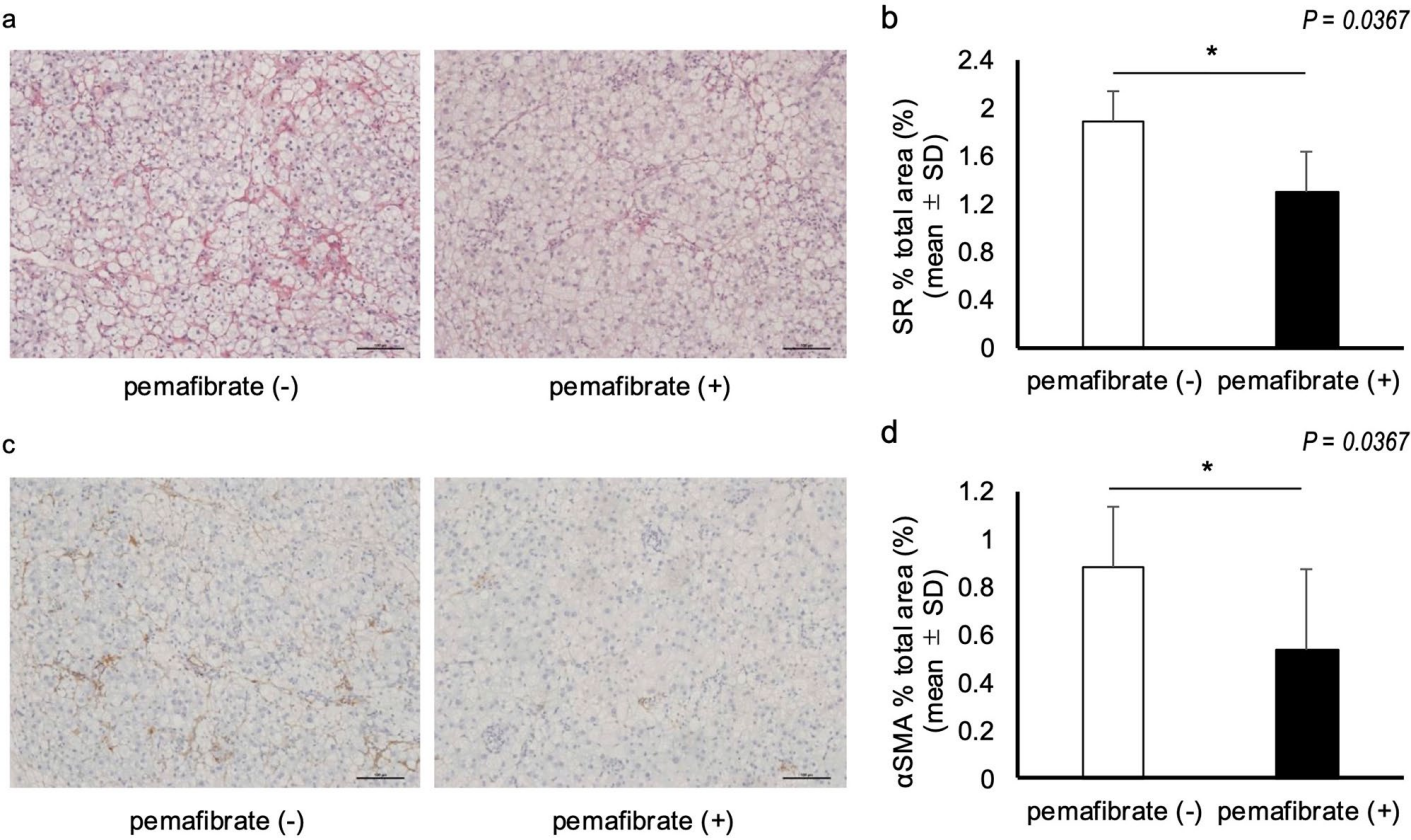
Pemafibrate improves liver fibrosis. (**a**) Liver sections from the pemafibrate (-) and pemafibrate (+) groups. Sirius Red staining. Magnification, 200×. Scale bar: 100 μm. (**b**) Area of Sirius red staining in the livers of rats from the pemafibrate (-) and pemafibrate (+) groups. *N* = 5 per group; *P < 0.05. (**c**) Liver sections of rats from the pemafibrate (-) and pemafibrate (+) groups. Anti-α-SMA antibody. Magnification, 200×. Scale bar: 100 μm. (**d**) Area of anti-α-SMA antibody in the livers of rats from the pemafibrate (-) and pemafibrate (+) groups. *N* = 5 per group; *P < 0.05. Data in the bar plots are expressed as mean ± standard deviation. Significant differences were determined using the Mann–Whitney U-test

The rats in the pemafibrate-treated group were administered pemafibrate (0.1 mg/kg/day) and olive oil (1 g/kg/day) by oral gavage for 7 days before being dissected. The pemafibrate-untreated group (control) rats received vehicle instead of pemafibrate while maintaining the same conditions mentioned above (Fig. 2a). The mRNA expression of *Ppara* was upregulated in the small intestine of rats in the pemafibrate-treated group compared with that in the pemafibrate-untreated group (*P* = 0.0304; Fig. 2b). We also evaluated the expression of FA absorption- and CM synthesis-related proteins and genes in the small intestine of these MASH model rats. The mRNA expression of *Cd36*, which encodes a scavenger receptor critical for the high-affinity uptake of long-chain fatty acids (LCFAs) and has been implicated in lipid accumulation and metabolic dysfunction when exposed to excessive fat [26], was significantly lower in the pemafibrate-treated group than in the pemafibrate-untreated group (*P* = 0.0304; Fig. 2c). Moreover, the mRNA level of *Mtp*, which encodes a rate-limiting enzyme involved in CM formation and the incorporation of TG into CM in the endoplasmic reticulum [27, 28], was significantly lower in the pemafibrate-treated group than in the pemafibrate-untreated group (*P* = 0.0304; Fig. 2d). Fatty acid-binding proteins (FABPs) are cytosolic proteins that bind to LCFAs and transport FAs in the cytoplasm. Two FABP subtypes are expressed in the intestine: liver (L)-FABP and intestinal (I)-FABP [29]. The mRNA expression of *Fabp1* (L-FABP) was significantly lower in the pemafibrate-treated group than in the pemafibrate-untreated group (*P* = 0.0304; Fig. 3e). The mRNA expression of *Fabp2* (I-FABP) was also lower in the pemafibrate-treated group than in the pemafibrate-untreated group, but the difference was not significant (*P* = 0.112; Fig. 2f). Moreover, no significant differences in the mRNA expression levels of fatty acid transport protein 4 (*Fatp4*), which indirectly drives FA uptake by esterification [30], and apolipoprotein A-IV (*Apoa-IV*), which encodes the major apoprotein component of chylomicrons, were observed between the groups (*P* = 0.471, *P* = 0.194; Fig. 2g and h).

These results suggest that pemafibrate inhibits the mRNA expression levels involved in LCFA absorption and transport, such as CD36 and MTP, in MASH model rats.

### Effect of pemafibrate treatment on hepatic fibrosis and hepatic stellate cell activation

We assessed fat deposition and liver fibrosis in both groups via H&E staining, Sirius Red staining, and immunostaining for α-SMA in the liver tissues with the same protocol described above (Fig. 2a). We then performed real-time PCR analysis of genes related to hepatic stellate cell activation. No significant difference in the degree of fat deposition was observed between the groups (data not shown). However, pemafibrate significantly improved liver fibrosis (*P* = 0.0367; Fig. 3a and b). Moreover, the number of α-SMA-positive cells was significantly lower in the pemafibrate-treated group than in the pemafibrate-untreated group (*P* = 0.0367; Fig. 3c and d).

**Fig. 4:**
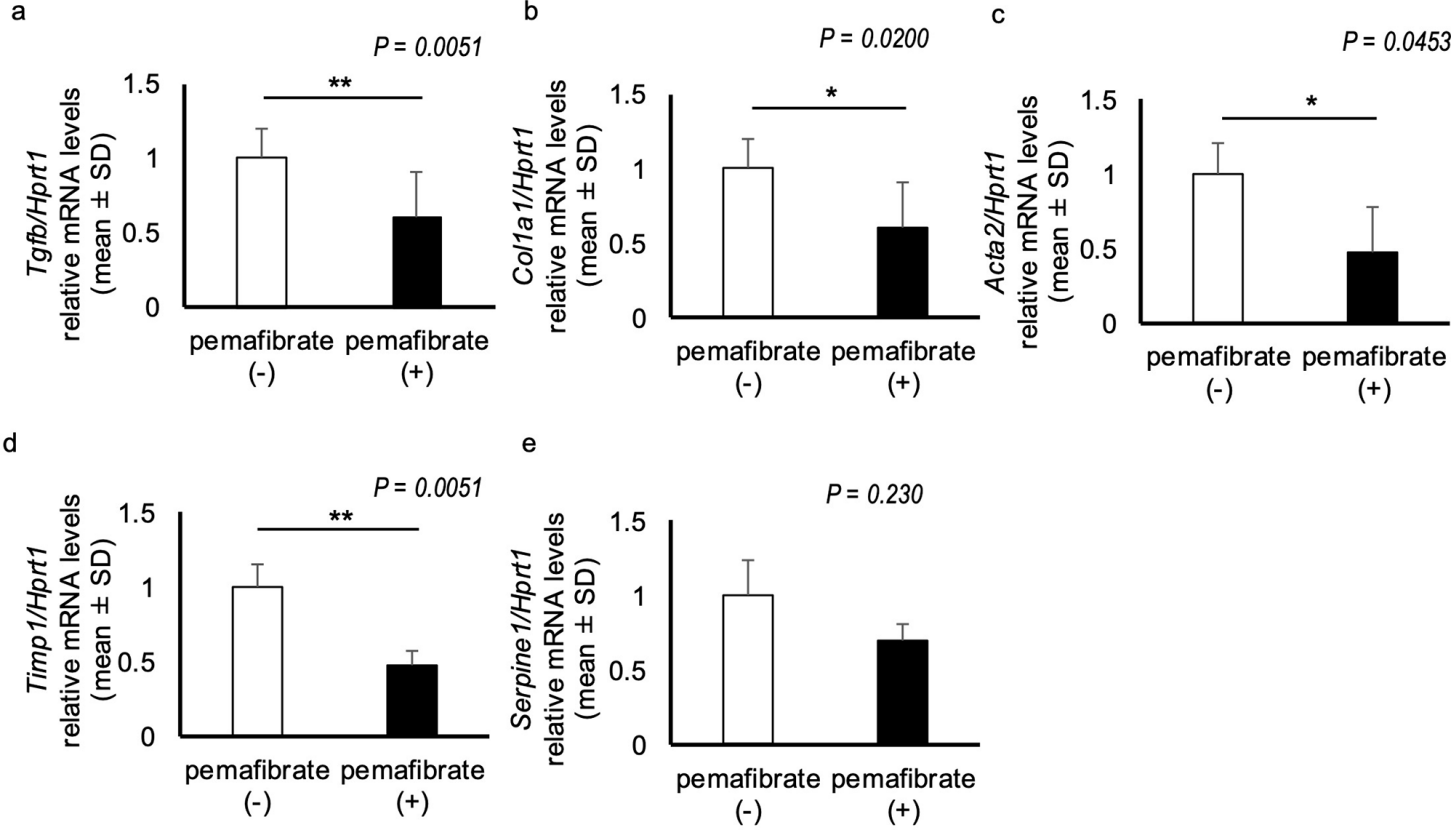
Expression of genes involved in fibrosis and HSC activation in the liver. (**a**) mRNA expression levels of liver fibrosis markers, transforming growth factor-β (*Tgfb*), (**b**) collagen 1a1 (*Col1a1*), (**c**) α-smooth muscle actin (*Acta2*), (**d**) tissue inhibitor of metalloproteinase 1 (*Timp1*), and (**e**) plasminogen activator inhibitor 1 (*Serpine1*). *N* = 5 per group; *P < 0.05. **P < 0.01. Data in the bar plots are expressed as mean ± standard deviation. Significant differences were determined using the Mann–Whitney U-test

Transforming growth factor-β (TGF-β) activates HSCs [31]. Notably, the mRNA expression of TGF-β (*Tgfb*) was significantly lower in the pemafibrate-treated group than in the pemafibrate-untreated group (*P =* 0.0051; Fig. 4a). The mRNA expression of collagen 1a1 (*Col1a1*) and α-SMA (*Acta2*) was also decreased via the inhibition of hepatic stellate cell activation (*P* = 0.0200, *P* = 0.0453, respectively; Fig. 4b and c). Tissue inhibitor of metalloproteinase (TIMP) is secreted by activated HSCs and is involved in fibrosis by inactivating matrix metalloproteinase [32]. The mRNA expression of *Timp1* was significantly lower in the pemafibrate-treated group than in the pemafibrate-untreated group (*P* = 0.0051; Fig. 4d). Plasminogen activator inhibitor 1 (PAI-1) promotes fibrosis in many tissue types [33]. The mRNA expression of PAI-1 (*Serpine1*) was lower in the pemafibrate-treated group than in the pemafibrate-untreated group, although the difference was not significant (*P* = 0.230; Fig. 4e).

These results suggest that treatment with pemafibrate reduces hepatic stellate cell activation and hepatic fibrosis in MASH model rats.

## Discussion

In the present study, we demonstrated that treatment with pemafibrate inhibited intestinal lipid absorption in a rat model of diet-induced MASH. In addition, pemafibrate downregulated the expression of LCFA receptors and chylomicron synthesis-related molecules in the small intestine. Although pemafibrate has been reported to improve liver fibrosis in MASH models [24, 34], the results of the present study suggest that not only direct hepatic effects but also suppression of intestinal FA absorption may be partially responsible for the improvement of fibrosis in MASH. Pemafibrate has been reported to suppress intestinal cholesterol absorption [35], but this study, to our knowledge, is the first to conduct a histological evaluation of the effects of a pemafibrate on small intestinal LCFA absorption and lipid droplet synthesis in an MASH model in vivo.

A previous study reported that feeding SD rats a high-fat diet for a short period increases the amounts of triglycerides and total FAs in the liver by approximately three-fold but does not significantly increase the amounts of visceral fat and skeletal muscle fat [36]. This finding suggests that a high-fat diet may promote fat accumulation in the liver. In addition, several studies have reported alterations in the kinetics of dietary lipid absorption in patients with MASLD. Oral lipid loading experiments have indicated that the serum triglyceride response after lipid loading is significantly higher in patients with fatty liver than in healthy individuals [37]. Moreover, the small intestinal absorption capacity of palmitic acid is significantly higher in patients with MASH than in healthy individuals, and the amount of dietary palmitate absorbed positively correlates with liver fibrosis and insulin resistance [13]. Proteins associated with intestinal FA absorption, such as glycosylated CD36 and MTP, were overexpressed in small intestinal biopsy tissues collected from patients with MASH [13] and model rats [14], highlighting a potential mechanism for increased dietary palmitic acid absorption in MASH progression. In addition, we observed a significant downregulation of intestinal Cd36 mRNA expression by pemafibrate treatment (Fig. 2c). In the intestinal epithelium, the primary function of CD36 is the uptake of dietary LCFAs. PPARα activation has been reported to upregulate CD36 expression in other cell types, such as macrophages and hepatocytes, often linked to fatty acid uptake for metabolism or storage [38]. It is possible that in the context of systemic lipid overload induced by the HFHCD, PPARα activation serves an adaptive role by downregulating the expression of this key lipid transporter in the intestine to limit further substrate influx, differing from its role in other tissues. Furthermore, the HFHCD likely influences CD36 expression independently and complicates the net effect of pemafibrate. Further studies are warranted to dissect the precise molecular mechanisms underlying this context-dependent regulation.

The role of intestinal PPARα in lipid homeostasis is largely unknown, but pemafibrate, a selective PPARα modulator, increases the expression of PPARα and genes related to FA oxidation and decreases cholesterol absorption in the small intestine [39]. In the present study, treatment of a rat MASH model with pemafibrate significantly increased the mRNA levels of intestinal *Ppara* and decreased the expression of molecules involved in intestinal lipid regulation. Moreover, triglyceride deposition and lipid absorption were significantly reduced in the intestinal tissues. These findings suggest that small intestinal PPARα is a potential therapeutic target for MASH. In contrast, a recent study has suggested that intestinal PPARα signaling promotes MASH progression by regulating dietary FA uptake through induced FABP1 expression [40]. These conflicting results are potentially attributable to differences in animal models. Moreover, we evaluated intestinal lipid absorption in vivo using pemafibrate; in the previous study, it was evaluated in vitro using intestinal organoids. Nevertheless, further human studies are warranted for verification.

In the present study, pemafibrate had a notable effect on hepatic fibrosis, a cardinal feature of MASH. It considerably improved hepatic fibrosis in the pemafibrate-treated group, as indicated by the reduced number of α-SMA-positive cells, a marker of hepatic stellate cell activation [41]. Moreover, treatment with pemafibrate decreased the mRNA expression of fibrosis-related genes, such as *Tgfb*, *Col1a1*, and *Acta2*. This result is consistent with the findings of previous studies suggesting that PPARα agonists can ameliorate hepatic fibrosis by acting directly on hepatic PPARα [35, 42]. Moreover, we previously reported that increased fat absorption from the intestinal tract affects liver fibrosis in MASH [14]. The present study suggests that pemafibrate inhibits liver fibrosis by suppressing fat absorption from the intestinal tract.

This study has some limitations. First, a rat model of MASH was used. While the high-fat, high-cholesterol diet (HFHCD)-induced rat model employed in this study recapitulates key pathological features of human MASH, including hepatic steatosis, inflammation, and fibrosis, making it a valuable tool for investigating lipid metabolism and fibrogenesis [25], inherent limitations must be acknowledged when extrapolating these findings to the human condition. The complex pathophysiology of human MASH, often driven by multiple concurrent factors (‘multiple parallel hits’) [43]. Furthermore, potential species-specific variations in lipid metabolism, dietary responses, and the precise downstream consequences of PPARα activation exist between rodents and humans. Therefore, although our study provides important preclinical mechanistic insights into pemafibrate’s dual effects on intestinal lipid handling and hepatic fibrogenesis in this model, direct translation of the magnitude and therapeutic implications of these effects requires considerable caution. Translating these preclinical findings requires clinical context. While pemafibrate, used clinically for dyslipidemia, was associated with reduced MRE-measured liver stiffness in MASLD patients [44], direct histological evidence of anti-fibrotic effects in humans is absent. Crucially, clinical data on pemafibrate’s effects on human intestinal lipid absorption pathways are currently lacking, representing a key knowledge gap. Moreover, human MASLD/MASH is a chronic disease developing over years, suggesting that the short 7-day treatment duration used in our study, while sufficient to observe initial mechanistic changes in gene expression and lipid markers based on non-clinical pharmacodynamic data, is likely insufficient to predict long-term therapeutic efficacy on established fibrosis. Consequently, extrapolating direct therapeutic potential, particularly for fibrosis reversal, from this short-term study warrants caution. Nevertheless, our findings demonstrating concurrent modulation of intestinal lipid handling markers and hepatic fibrosis markers provide a strong rationale for further investigating pemafibrate in human MASLD/MASH, potentially as an adjunct therapy. Future long-term clinical trials are needed to assess efficacy and safety. Such trials could explore combination strategies, for example, pairing pemafibrate with inhibitors of intestinal cholesterol absorption like ezetimibe, to potentially achieve synergistic effects on both the metabolic drivers and the progression of hepatic fibrosis in patients.

Second, in this study, we did not establish a healthy control group. Hence, our ability to evaluate the severity of the parameters in the intestinal tract and liver induced by MASH is limited; moreover we cannot determine the extent to which improvement due to pemafibrate administration contributes to normalization when compared with a healthy control group. As a reference, in our previous study [14], we confirmed that the expression of genes related to intestinal LCFA absorption (CD36, Mtp, and Fabp1) is increased by approximately 1.5 times higher in MASH model rats than in normal healthy control (normal diet) rats. In the present study, the expression of these genes was significantly suppressed by pemafibrate administration (approximately 40% for CD36 and approximately 20% for Mtp and Fabp1). These results indicate that pemafibrate treatment significantly reduces the severity of MASH-induced intestinal LCFA absorption abnormalities; however, the Mtp and Fabp1 levels in the MASH may still be higher than those in the healthy control levels, and complete normalization may not be achieved. Also in this study, we were unable to show complete equivalence between the two groups at the start of the intervention. However, we tried to eliminate bias between the groups as much as possible by randomly assigning rats inducing MASH to the pemafibrate-treated group and the pemafibrte-untreated group. Although our data suggest that pemafibrate treatment reduces intestinal LCFA absorption and hepatic fibrosis in parallel, the study design does not allow us to establish a direct causal relationship. Pemafibrate, as a systemic PPARα agonist, may exert independent hepatic effects, complicating the attribution of fibrosis improvement solely to reduced intestinal lipid uptake. Future studies should employ intestinal-specific PPARα modulation or liver-specific knockout models will be essential to delineate the mechanistic pathways.

Third, the main lipid-processing molecules, including CD36, have not been verified at the protein level. We observed a significant decrease in lipid metabolism factors at the mRNA level, but this does not necessarily mean that we can evaluate their activity. Similarly, analysis of the protein levels of typical activation markers of PPARα in the liver was not conducted in this study. It has been well established in other studies that pemafibrate actually activates PPARα in the liver and increases the expression of genes involved in fatty acid oxidation, such as CPT1A and ACOX1 [24, 45], and we did not measure the expression of these classic PPARα target genes. The observed beneficial phenotype and upregulation of Ppara mRNA itself in the gut suggest that pemafibrate acted through PPARα, but the lack of data confirming the induction of these classic upregulation target genes is a limitation in terms of providing direct molecular-level evidence of pathway activation in this model.

Forth, the sample size in this study was relatively small. While this reflects the exploratory nature of the study and adherence to the 3R’s principle in animal experimentation, it may limit statistical power. Consequently, the risk of false negatives (Type II errors) might be increased, particularly for parameters with smaller effect sizes or larger inter-individual variability. Indeed, some parameters, such as intestinal Fabp2 and PAI-1 mRNA expression, did not show statistically significant differences between the groups in our study. This lack of significance could potentially be due to insufficient statistical power, and thus, these non-significant findings should be interpreted with caution. Future studies with larger sample sizes are recommended to confirm the main effects observed in this study and enhance the generalizability of the findings.

Fifth, the reduction in lipid accumulation observed through Oil Red O staining appeared significantly greater than the observed reduction in overall fat absorption. This discrepancy suggests that the reduction in staining may not solely indicate reduced lipid absorption but may also reflect enhanced lipid oxidation or altered intracellular lipid trafficking within the epithelial cells. Following pemafibrate administration, lipid mobilization and utilization may be enhanced, resulting in a more rapid clearance of intracellular lipid droplets despite minimal changes in the net fat absorption. This dynamic change in lipid metabolism could explain the mismatch between staining reduction and fat absorption rates.

As described above, this study focused mainly on intestinal lipid processing and hepatic fibrosis, but a systematic analysis of a wide range of metabolic parameters was not conducted. Therefore, although the effects on specific endpoints were observed, the effects of pemafibrate on systemic metabolism in this model have not been comprehensively evaluated, indicating the limitations of this initial investigation and requiring further research.

## Conclusions

Treatment of a rat model of MASH with pemafibrate reduced intestinal lipid droplet synthesis and absorption, decreased hepatic stellate cell activation, and inhibited hepatic fibrosis. These results demonstrate the dual actions of pemafibrate in reducing intestinal LCFA absorption and ameliorating hepatic fibrosis, suggesting its potential as a therapeutic agent for MASH. This study provides novel insights into the effects of a PPARα agonist on intestinal LCFA absorption and hepatic fibrosis in MASH model rats.

## Data Availability

The datasets used and/or analysed during the current study are available from the corresponding author on reasonable request.

## Abbreviations

ApoA-IV: Apolipoprotein A-IV
α-SMA: α-smooth muscle actin
CD36: Cluster of differentiation 36
CM: Chylomicron
Col1a1: Collagen1a1
FABP: Fatty acid-binding protein
FATP4: Fatty acid transport protein 4
HSC: Hepatic stellate cell
H&E: Hematoxylin and eosin
LAC: Lipid absorption coefficient
LCFA: Long-chain fatty acid
MTP: Microsomal triglyceride transfer protein
MASH: Metabolic dysfunction-associated steatohepatitis
MASLD: Metabolic dysfunction-associated steatotic liver disease
NAFLD: Non-alcoholic fatty liver disease
NASH: Non-alcoholic steatohepatitis
PAI-1: Plasminogen activator inhibitor 1
PPAR: Peroxisome proliferator-activated receptors
SFA: Saturated fatty acids
TG: Triglyceride
TGF-β: Transforming growth factor-β
TIMP: Tissue inhibitor of metalloproteinase
